# Risk factors for venous thromboembolism of total hip arthroplasty and total knee arthroplasty: a systematic review of evidences in ten years

**DOI:** 10.1186/s12891-015-0470-0

**Published:** 2015-02-10

**Authors:** Zi-hao Zhang, Bin Shen, Jing Yang, Zong-ke Zhou, Peng-de Kang, Fu-xing Pei

**Affiliations:** Department of Orthopaedics surgery, West China Hospital of Sichuan University, Chengdu, Sichuan P.R. China

**Keywords:** Total hip arthroplasty, Total knee arthroplasty, Venous thromboembolism, Risk factor, Systematic review

## Abstract

**Background:**

Risk factors for venous thromboembolism (VTE) of total joint arthroplasty (TJA) have been examined by many studies. A comprehensive systematic review of recent findings of high evidence level in this topic is needed.

**Methods:**

We conducted a PubMed search for papers published between 2003 and 2013 that provided level-I and level-II evidences on risk factors for VTE of TJA. For each potential factors examined in at least three papers, we summarize the the number of the papers and confirmed the direction of statistically significant associations, e.g. “risk factor” “protective factor” or “controversial factor”.

**Results:**

Fifty-four papers were included in the systematic review. Risk factors found to be associated with VTE of both total hip arthroplasty and total knee arthroplasty included older age, female sex, higher BMI, bilateral surgery, surgery time > 2 hours. VTE history was found as a VTE risk factor of THA but an controversial factor of TKA. Cemented fixation as compared to cementless fixation was found as a risk factor for VTE only of TKA. TKA surgery itself was confirmed as a VTE risk factor compared with THA surgery.

**Conclusions:**

This systematic review of high level evidences published in recent ten years identified a range of potential factors associated with VTE risk of total joint arthroplasty. These results can provide informations in this topic for doctors, patients and researchers.

## Background

Venous thrombpembolism (VTE) remains a problem in patients after undergoing the total joint arthroplasty(TJA), which includes total hip arthroplasty (THA) and total knee arthroplasty (TKA) [1-4]. Among in-hospital patients who received recommended VTE prophylaxis, symptomatic deep vein thrombosis (DVT) rates were 0.26%-0.63% and rates for pulmonary embolism (PE) were 0.14%-0.27% after total joint arthroplasty, reported by a systematic review [5]. Patients are suffering from 10 times of healthcare costs and more than twice of length staying in hospital compared with those without VTE, and the mortality rate associated with pulmonary embolism(PE) is reported to be 19.49% [6]. In consideration of the large number of TJA patients worldwide, VTE remains threatening.

Close monitoring regime and appropriate thromboprophylaxis are in urgent need to minimize the rate of VTE, which should be based on VTE risk stratification. Identifying VTE risk factors is crucial and challenging because there are a number of potential VTE risk factors being worthy of note. Several individual studies [7] focusing on specific risk factors such as previous thrombosis, malignancy and so forth represent a potential wealth of evidence regarding a range of VTE risk factors after TJA. A table of “commonly cited risk factors” for VTE after total joint arthroplasty provided by AAOS [8] can be applied to clinical practice in risk identifying. However, there have been no efforts to aggregate existing reservoir of evidence of a more comprehensive set of risk factors, to our knowledge.

To summarize the published literature on risk factors for VTE of TJA relating to patient demographic and clinical factors, laboratory indexes, health care provider characteristics and thromboprophylaxes, we conducted this systematic review of high level studies [9] in recent ten years (2003–2013). This effort may be helpful to improve our knowledge and therefore promote a new VTE risk assessment system for patients undergoing total joint arthroplasty and doctors paying attention to this issue.

## Methods

### Search strategy

We conducted a PubMed search on May 26th, 2013 to identify studies published between January 1st, 2003 and May 1st, 2013. There is no restriction of language or country. The search strategy was designed and peer-reviewed before the beginning of search, which is listed as follows:

((((thrombosis[Title/Abstract]) OR thromboembolism[Title/Abstract]) OR embolism [Title/Abstract])) AND ((((risk factor) OR risk factors)) AND ((((total hip arthroplasty [Title/Abstract]) OR total hip replacement [Title/Abstract]) OR total knee arthroplasty [Title/Abstract]) OR total knee replacement [Title/Abstract])).

Screening has been performed sequentially in three levels: title, abstract and then full-text as recommended by the PRISMA Statement criteria [10]. The exclusion criteria is outlined in Table 1. To guarantee the strength of evidence, only level-I and level-II prognostic studies were included. Specifically, High-quality prospective studies in which all patients were enrolled at the same point in their disease with ≥80% follow-up of enrolled patients are taken as the level-I, whereas retrospective studies, untreated controls from a randomized controlled trial and lesser-quality prospective studies (e.g., patients enrolled at different points in their disease or <80% follow-up) are seen as the level-II. In this way, we assessed the risk of bias of included studies mainly based on their study types and follow-up rates which can be summed up as the “evidence level”. Also, we assessed all the included papers using the Newcastle-Ottawa Scale (NOS) of which the detail can be found at the website: http://www.ohri.ca/programs/clinical_epidemiology/oxford.htm.

**Table 1:** **Exclusion criteria**

Exclusion criteria	Example or explanation
No living human subjects	*e.g. mice model*
Focus on wrong procedure	*e.g. unicompartmental knee replace*-*ment*
Focus on risk factors for other complications	*e.g. limb swelling*
Focus on other thrombo-related factors	*e.g. bleeding or blood transfusion*
Focus on fat embolism	*e.g. fat embolism after femoral head resection*
Have not mentioned any risk factor	*e.g. prevelance studied alone*
Not primary clinical research	*e.g. literature review or guidline*
Level III prognostic study	*e.g. case*–*control*
Level IV prognostic study	*e.g. case series*
Level V prognostic study	*e.g. expert opinion*
Diagnostic study	*e.g. spiral CT for the detection of pulmonary embolism*
Economic and decision analyses	*e.g. cost*-*effectiveness of extended*-*duration thromboprophylaxis after THA*
Indispensable data missing or not available	*e.g. no p*-*value*
Article could not be retrieved	*e.g. not available in electronic or print archives*
Duplicate publication	*e.g. Similar title*, *sample size* ,*and outcome data*

### Validation

Eligibility assessment was performed independently in an unblinded standardized manner by 2 reviewers (the first and the second author). Disagreements between reviewers were adjudicated by the senior authors (including the third to the sixth author) and ultimately resolved by consensus.

### Data abstraction

Data were extracted independently by 2 reviewers from the included articles. We extracted information on country, study design, sample size, follow-up time and follow-up rates. in this process., We also make distinction between the VTE risk factors for THA and for TKA. Effect measures such as risk ratio, odds ratio were collected whenever available, as well as the p-values. Corresponding authors of the included articles were contacted for detailed information or numerical data, if needed. All of the extractions are based on a preformed sheet.

### Analysis

A formal meta-analysis can not be done because of the heterogenous nature among the studies’ type (prospective and retrospective), follow-up times(from less than 1 week to 3 years) and risk factor specifications. Instead, we identified all the VTE risk factors reported in at least one high level study and the number of reporting studies, then described the direction of significant associations(defined as p ≤ 0.05 or confidence intervals which are non-overlapping) of risk factors reported by at least 3 studies. To categorize the included studies in this way may have limitations because this approach does not measure the heterogeneity among the studies. However, It does build up a framework of the state across the recent literatures.

As a basic step, the correlations between the risk factors (or probably the protective factors) and VTE were classified into three categories: “p ≤ 0.05,+” “p ≤ 0.05,-” and “p > 0.05”, which means “a significant increased risk (risk factor)” “a significant decreased risk (protective factor)” and “with no significant association (controversial factor)”, respectively.

In the second step, we made comparisons and determined the direction of significant associations for each of the factors according to the following rules:

1.A factor reported with “p > 0.05” by all papers focusing on it is defined as the “*controversial factor*”;
2.For a factor reported with no fewer than 2 of the 3 categories, e.g. “+”and“-”, as the results, the proportion number of the first category(“+”) to that of the second category(“-”) will be compared and the winner was taken as the final result. In this way, the factor is defined as the “*risk factor*” or “*protective factor*”.

Therefore we *confirmed* the VTE risk factors of total joint arthroplasty.

## Ethical compliance

This study was a data-based systematic review and was not performed on humans. In this way, no ethical approval was required.

## Results

### Screening results

We included 54 papers [11-64] from 226 identified titles, in which about 1,150,000 patients from more than 30 countries and 28 classes of factors were examined. All the included studies were above level-II and 10 of them (19%) were level-I. The NOS results were provided in Table 2. Twenty-eight classes of factors were examined. The overall frequences of studies and reference numbers were collected (see Tables 3 and 4). We summarize all factors examined by at least one study in Tables 4, 5, 6, 7, 8 and 9, in which the factors were classified into five aspects: demographic factors, clinical factors, laboratory indexes, health care provider-related factors and thromboprophylaxes. The factor examined by at least three articles are qualified to be determined whether it is a “risk factor”, a “protective factor” or an “controversial factor” (see Table 10). Factors examined by fewer than three articles were included but not discussed.

**Table 2:** **NOS of the included studies**

NOS	Percentages of studies N(n%)*	References of studies
8☆	14 (26%)	[11,14,27,32,38,39,41,43,45,48,50,54,56,63]
7☆	22 (41%)	[15,18,20-23,26,29,35-37,47,49,51,52,55,57-62,64]
6☆	18 (33%)	[12,13,16,17,19,24,25,28,30,31,33,34,40,42,44,46,53]

*N (n%), N = number of studies, n = percentage in the 54 included studies.

**Table 3:** **Number of studies for potential factors**

Endpoint	VTE*
Total of the endpoint	54
**Risk factors**	**N** ^**†**^ **(%)**
**Demographic factors**	
Age	12 (22%)
Gender	13 (24%)
BMI	6 (11%)
Race	1 (2%)
ASA physical status	3 (6%)
**Clinical factors**	
Underlying diagnosis	3 (6%)
Comorbidity (Charlson index)	3 (6%)
Cardiovascular disease	10 (19%)
Respiratory disease	2 (4%)
Neurological disease	1 (2%)
Liver and kidney disease	2 (4%)
Metabolic disease	7 (13%)
Hematological disease	2 (4%)
Endocrine disease	2 (4%)
Malignancy	4 (8%)
Medication (hormone replacement/herbal)	3 (6%)
**Laboratory indexes**	
Preoperative laboratory index	2 (4%)
Postoperative laboratory index	2 (4%)
**Health care provider-** **related factors**	
Surgery type	12 (22%)
Surgical technic	5 (9%)
Operating time	4 (8%)
Anesthesia	5 (9%)
Bleeding	1 (2%)
Hospital volume	2 (4%)
Insurance type	1 (2%)
**Thromboprophylaxes**	
Chemoprophylaxis	28 (52%)
Initiating and lasting of the prophylaxis	4 (8%)
Mechanical and physical prophylaxis	5 (9%)

*VTE = venous thromboenbolism,which includes *DVT*(deep vein thrombosis) and *PE* (pulmonary embolism).

^†^Column percentages,not mutually exclusive.

**Table 4:** **References of studies for potential factors**

Risk factors	Reference number’s for papers
**Demographic factors**	
Age	[22,23,26,28-30,35,41,47,52,54,63]
Gender	[18,22,23,26,28-30,35,41,47,52-54]
BMI	[26,28-30,35,54]
Race	[23]
ASA physical status	[35,52,54]
**Clinical factors**	
Underlying diagnosis	[26,46,47]
Comorbidity (Charlson index)	[23,47,54]
Cardiovascular disease	[18,22,26,29,30,35,47,53,54,62]
Respiratory disease	[35,58]
Neurological disease	[35]
Liver and kidney disease	[47,61]
Metabolic disease	[26,35,37,47,53,59,64]
Hematological disease	[26,35]
Endocrine disease	[26,35]
Malignancy	[26,35,47,53]
Medication (hormone replacement/herbal)	[18,26,29]
**Laboratory indexes**	
Preoperative laboratory index	[35,49]
Postoperative laboratory index	[28,53]
**Health care provider-** **related factors**	
Surgery type	[11,13,18,20,21,26,27,29,30,35,41,53]
Surgical technic	[12,13,18,21,29]
Operating time	[19,22,35,49]
Anesthesia	[18,21,29,30,52]
Bleeding	[28]
Hospital volume	[23,55]
Insurance type	[23]
**Thromboprophylaxes**	
Chemoprophylaxis	[14-18,21,25,26,28,31-34,36,38-40,42-45,47,48,50,51,56,60,61]
Initiating and lasting of the prophylaxis	[21,30,43,57]
Mechanical and physical prophylaxis	[19,24,29,30,52]

**Table 5:** **Demographic factors**

Risk factors(demographic factors)	Studies reporting on a risk factor for THA or TKA:total number,number reporting a significant (p ≤ .05)increased(+)or decreased (−) risk,and number with no significant association (p > .05)							
	THA				TKA			
	N	p ≤ .05		p > .05	N	p ≤ .05		p > .05
		+	-			+	-	
Older age	8	6	0	2	9	3	0	6
Female sex	9	2	1	6	9	2	0	7
Higher BMI*	3	1	0	2	6	1	0	5
Black race (vs. white)	-	-	-	-	1	1	0	0
Hispanic race (vs. white)	-	-	-	-	1	0	0	1
Higher ASA score^†^	3	0	0	3	2	0	0	2

*BMI = Body Mass Index.

^†^The ASA (American Society of Anesthesiologists) score refers to the classification of the physical status of a patient before surgery.

**Table 6:** **Clinical factors**

Risk factors(clinical factors)	Studies reporting on a risk factor for THA or TKA:total number,number reporting a significant (p ≤ .05) increased (+)or decreased (−)risk,and number with no significant association (p >. 05)							
	THA				TKA			
	N	p ≤ .05		p > .05	N	p ≤ .05		p > .05
		+	-			+	-	
**Underlying diagnosis**								
RA (vs. without RA)	-	-	-	-	1	1	0	0
RA (vs. OA)*	1	0	1	0	1	0	1	0
Trauma (vs. OA)	1	0	0	1	-	-	-	-
Osteonecrosis (vs. OA)	1	0	0	1	-	-	-	-
Dysplasia (vs. OA)	1	0	0	1	-	-	-	-
**Comorbidity**								
Higher Charlson index^†^	2	0	0	2	2	1	0	1
Cardiovascular disease	1	1	0	0	-	-	-	-
Stroke	1	0	0	1	1	1	0	0
Heart disease	1	0	0	1	2	0	0	2
CHF/MI^‡^	2	1	0	1	1	1	0	0
Coronary artery disease	1	0	0	1	1	0	0	1
Valve disease	1	0	0	1	1	0	0	1
Arrhythmia	2	0	0	2	2	0	0	2
VTE history	6	2	0	4	4	0	0	4
Venous stasis	-	-	-	-	1	0	0	1
Varicose vein	2	1	0	1	3	0	0	3
Pulmonary disease	1	0	0	1	1	0	0	1
Sleep apnea	2	1	0	1	2	1	0	1
Neurological disease	1	0	0	1	1	0	0	1
Liver and kidney disease	1	0	0	1	-	-	-	-
CKD3B (vs. CKD1-3A)^§^	1	1	0	0	-	-	-	-
Metabolic syndrome	1	1	0	0	2	2	0	0
Diabetes mellitus	4	0	0	4	5	0	0	5
Hypertension	2	0	0	2	3	0	0	3
Dyslipidemia	2	0	0	2	2	0	0	2
Gout	-	-	-	-	1	0	0	1
Hematological disease	1	0	0	1	2	1	0	1
Endocrine disease	1	0	0	1	2	0	0	2
Malignancy	4	0	0	4	4	0	0	4
**Medication**								
Hormone replacement	1	0	0	1	3	0	0	3
Herbal therapy	-	-	-	-	1	1	0	0

*RA = rheumatoid arthritis, OA = osteoarthritis. ^†^Charlson inex refers to the classification of the comorbidity of a patient before surgery. ^‡^CHF = congesive heart failure, MI = Myocardial infarction. ^§^CKD = chronic kidney disease.

**Table 7:** **Laboratory indexes**

Risk factors(laboratory indexes)	Studies reporting on a risk factor for THA or TKA:total number,number reporting a significant (p ≤ .05)increased (+) or decreased (−) risk,and number with no significant association(p > .05)							
	THA				TKA			
	N	p ≤ .05		p > .05	N	p ≤ .05		p > .05
		+	-			+	-	
**Preoperative index**								
blood glucose ≥ 200 mg/dl	1	1	0	0	1	1	0	0
Resting PaO2 < 75 mmHg	-	-	-	-	1	0	0	1
Resting PaCO2 ≥ 45 mmHg	-	-	-	-	1	1	0	0
RVSP ≥ 35 mmHg*	-	-	-	-	1	0	0	1
**Postoperative index**								
Higher platelet counts	-	-	-	-	1	1	0	0
Hemoglobin ≥ 10.5 g/dl	-	-	-	-	1	1	0	0
AaDO2 ≥ 34 Torr^†^	-	-	-	-	1	1	0	0
Seroconvertion of IgG-class HIT Antibody^‡^	-	-	-	-	1	1	0	0

*RVSP = right ventricular systolic pressure, referring to pulmonary hypertension.

^†^AaDO2 = alveolar-arterial oxygen gradient.

^‡^HIT = heparin-induced thrombocytopenia, which is a thromboembolic complication that can occur with unfractionated heparin (UFH) or low molecular weight heparin (LMWH).

**Table 8:** **Health care provider**-**related factors**

Risk factors(health care provider - related factors)	Studies reporting on a risk factor for THA or TKA:total number,number reporting a significant (p ≤ .05)increased(+) or decreased (−) risk,and number with no significant association (p > .05)							
	THA				TKA			
	N	p ≤ .05		p > .05	N	p ≤ .05		p > .05
		+	-			+	-	
TKA (vs. THA)	4	2	0	2	4	2	0	2
THA (vs. resurfacing)	1	1	0	0	-	-	-	-
Revision (vs. primary)	1	0	0	1	2	1	0	1
Bilateral (vs. unilateral)	3	1	0	2	4	2	0	2
Right side (vs. left side)	-	-	-	-	1	0	0	1
Cement (vs. cementless)	2	1	0	1	3	2	0	1
ROBODOC* (vs. traditional)	1	0	1	0	-	-	-	-
Longer surgery time	3	1	0	2	3	1	0	2
General anesthesia	3	0	0	3	3	0	0	3
Spinal anesthesia	-	-	-	-	1	0	0	1
Bleeding ≥ 1280 ml	-	-	-	-	1	1	0	0
Lower hospital volume	1	1	0	0	2	1	0	1
Insurance type^†^	1	0	0	1	-	-	-	-

*ROBODOC is a femoral milling system which excavates the femoral canal precisely and may reduce intraoperative pulmonary embolism during cementless THA.

^†^The comparisons are among private insurance, Medicare and Medicaid of USA.

**Table 9:** **Thromboprophylaxes**

Risk factors (thromboprophylaxes)	Studies reporting on a risk factor for THA or TKA: total number, number reporting a significant (p ≤ .05) increased (+) or decreased (−) risk, and number with no significant association (p > .05)							
	THA				TKA			
	N	p ≤ .05		p > .05	N	p ≤ .05		p > .05
		+	-			+	-	
Chemoprophylaxis (vs. no-prophylaxis)	3	0	2	1	3	0	2	1
Enoxaparin (vs. other LMWH)	3	0	1	2	3	0	1	2
Low-dose LMWH (vs. high-dose LMWH)	-	-	-	-	1	0	0	1
Preoperative LMWH (vs. Postoperative)	1	0	0	1	2	0	0	2
Oligosaccharides* (vs. LMWH)	2	0	2	0	1	0	0	1
Direct factor-Xa inhibitor (vs. LMWH)	5	0	4	1	5	1	2	2
Direct factor-II inhibitor (vs. LMWH)	2	0	1	1	2	1	0	1
Partial factor-VII inhibitor (vs. LMWH)	-	-	-	-	1	0	1	0
NSAIDS (vs. LMWH)	2	0	0	2	1	0	0	1
VKA (vs. NSAIDS)	-	-	-	-	1	1	0	0
ACCP-recommended prophylaxis (vs. others )	1	0	1	0	1	0	1	0
Extended prophylaxis (vs. short)^†^	1	0	1	0	2	0	2	0
Mechanical prophylaxis (vs. chemoprophylaxis)	1	0	0	1	-	-	-	-
Below-knee stockings (vs. up-knee)	-	-	-	-	1	0	0	1
Earlier mobilization	2	0	2	0	3	0	3	0
Weight bearing within 48 h	1	0	0	1	1	0	0	1

*Oligosaccharides include fondaparinux and SR123781A (a synthetic oligosaccharide).

^†^Extended/short prophylaxis has two kinds of definations:

1. Thromboprophylaxis continued to Day 30 ± 5/Day 10 ± 2;

2. Thromboprophylaxis lasting > 14d/<14d.

**Table 10:** **Factors addressed and confirmed by at least three papers**

	THA	TKA
**Risk factors for VTE**	1. Older age	1. TKA (vs. THA)
	2. Female sex	2. Older age
	3. Higher BMI	3. Female sex
	4. Bilateral surgery	4. Higher BMI
	5. VTE history	5. Bilateral surgery
	6. Surgery time > 2 hours	6. Cemented fixation
		7. Surgery time > 2 hours
**Protective factors for VTE**	1. Chemoprophylaxis for VTE*	1. Chemoprophylaxis for VTE*
	2. Enoxaparin (vs. other LMWH)	2. Enoxaparin (vs. other LMWH)
	3. Direct F-Xa inhibitor (vs. LMWH)	3. Direct F-Xa inhibitor (vs. LMWH)
		4. Earlier mobilization
**Controversial factors for VTE**	1. Diabetes mellitus	1. Diabetes mellitus
	2. Malignancy	2. Malignancy
	3. General anesthesia	3. General anesthesia
	4. ASA score	4. VTE history
		5. Varicose vein
		6. Hypertension
		7. Hormone replacement

*Compared with no-prophylaxis patients.

**Figure 1:**
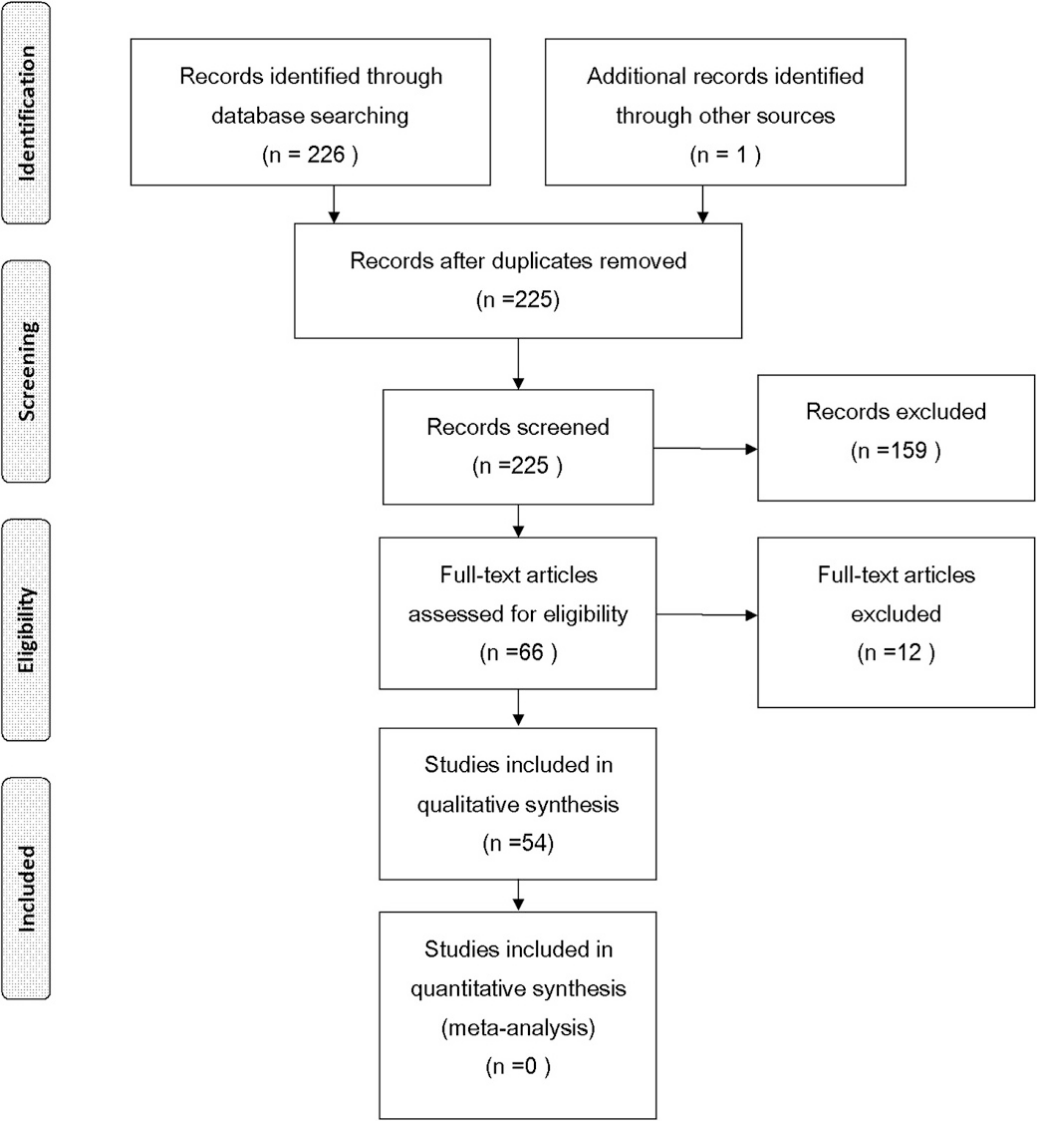
**Flow diagram of manuscript search and selection procedure.**

The full search screening procedure and results are presented in Figure 1. All but one [26] of the 54 papers have provided quantitative results. We contacted the corresponding author of that article by email and got an article published in his own country (Thailand) with sufficient supplementary data.

### Demographic factors

#### Age

Twelve papers [22,23,26,28-30,35,41,47,52-54] examined age as a risk factor for VTE. Both THA and TKA have got themselves reported in no fewer than eight papers. Of THA, six [22,23,30,35,41,52] reported an increased risk for older patients. Of TKA patients, three papers [23,30,35] reported older age as a risk factor for VTE.

A total of four papers [22,23,28,52] took “increased age” as the potential factor, while other papers investigated some specific cut-off age values such as 60, 70 ,80 and so on. Seniors older than 70 (vs. <70) or 75(vs. <75) were found with greater VTE risk [33,35]. However, the above results referring to the specific cut-off age values were constrained by the number of papers.

### Gender

Thirteen papers [18,22,23,26,28-30,35,41,47,52-54] examined gender as a risk factor for VTE. Both THA and TKA have got themselves reported in nine papers. Of THA, two [22,41] reported an increased risk for female patients while another study [22] found female gender with decreased VTE risk. Of TKA patients, two papers [53,54] reported female gender as a risk factor for VTE.

### BMI

Six papers [26,28-30,35,54] examined BMI as a risk factor for VTE. Of THA, one paper [35] among the total of three papers [30,35,54] reported an increased risk for patients with higher BMI. Of TKA patients, six papers examined BMI and one [35] found it a risk factor. The significant cut-off BMI value of either TKA or THA is 30. Patients with BMI higher than 30 have greater VTE risks than those with BMI less than 25.

### Race

One paper [23] examined race as a risk factor for VTE for TKA. The black race was found to be a risk factor while Hispanic race showed no significant difference when compared to the white race. Hispanic race was also investigated in the same paper but found with no significant association with VTE when comparing with the white race.

### ASA physical status

Three papers [35,52,54] examined ASA score as a risk factor for VTE. Three papers [35,52,54] for THA and two papers [35,54] for TKA examined this factor. Consistently, none of these papers reported significant association between ASA score and VTE incidence. Researchers of all the three papers used “ASA = 3 or 4” as the potential factor to compare with “ASA = 1 or 2” but found no significant difference.

### Clinical factors

#### Underlying diagnosis

Three papers examined underlying diagnoses, e.g. RA or OA, as risk factors for VTE. One paper [26] described RA as a risk factor for VTE of TKA. In one paper [46]of TKA and another paper of THA [47], researchers found RA a protective factor compared to OA. No significant association was found when referring to trauma, dysplasia and osteonecrosis.

### Comorbidity (charlson index)

Three papers examined Charlson comorbidity index as a risk factor and one paper [54] reported an increased risk for VTE of TKA patients. Charlson index is widely used to assess the severity of patients’ comorbidities before surgery and higher charlson scores indicate worse conditions. Some reseachers [23,47] used different cut-off values of charlson index, e.g. “1” “3” to compare with “0” value, while the other one paper [54] used “1 point increase” as the potential risk factor.

### Cardiovascular disease

Ten papers [18,22,26,29,30,35,47,53,54,62] examined cardiovascular diseases as a risk factors for VTE. Heart diseases, cerebrovascular diseases and venous disorders were included in this class. VTE history was found a significant risk factor for THA patients by two [22,47] in six papers, but with no association with VTE for TKA. Varicose vein was examined by three papers [26,30,62] focusing on TKA and turned out to be an controversial factor. The rest of the cardiovascular factors reported by less amount of papers were presented in Table 6.

### Respiratory disease

Respiratory diseases including pulmonary disease and sleep apnea were examined by two papers. Pulmonary disease is found with no association with VTE for either THA or TKA. One [58] from two papers [35,58] reported an increased risk of VTE for sleep apnea, applicable to both THA and TKA.

### Neurological disease

One paper [35] examined neurological diseases as risk factors of VTE for THA and TKA. No significant association was found between VTE and both the two kinds of surgeries. No detail about the neurological disease type was presented in the paper.

### Liver and kidney disease

Two papers [47,61] examined liver and kidney diseases including chronic kidney disease (CKD) as risk factors of VTE. One study [61] found CKD3B a risk factor for THA patients who received enoxaparin as thromboprophylaxis, compared to milder CKDs, e.g. CKD1, CKD2 or CKD3A. In addition, researchers of the above paper have also took “CKD3B” as a potential risk factor for THA patients who received desirudin, but found no significant association between CKD and VTE rate.

### Metabolic disease

Six paper [26,35,37,47,53,59] examined metabolic diseases as a risk factors of VTE for THA and TKA. One paper [59] for THA and two papers [37,59] for TKA found metabolic syndrome with increased VTE risk.

Diabetes mellitus was examined by four studies [26,35,47,53] for both THA and TKA, and showed no significant association with VTE. Risk directions of hypertension [26,35,53], dyslipidemia [35,53] and gout [26] were presented in Table 6. The influences of confounders among the above metabolic diseases still remain unclear.

### Hematological disease

Two papers [26,35] examined hematological disease as a risk factor of VTE. One [26] paper focusing on TKA found hematological a VTE risk factor. No details about the specific types of the hematological diseases were presented in the above paper.

### Endocrine disease

Two papers [26,35] examined endocrine disease as a risk factor of VTE. One paper [35] for THA and two for TKA [26,35] found no significant association between endocrine disease and VTE. The specific types of the endocrine diseases were not stated except hypothyroid disorder in one article [26].

### Malignancy

Four papers [26,35,47,53] examined malignancy as a potential factor of VTE for THA and TKA but none of them reported a significant association for VTE. Neither the type nor the stage of the malignancy was mentioned in any papers.

### Medication (hormone replacement/herbal)

Three papers [18,26,29] examined medications including hormone replacement and herbal as risk factors. Hormone replacement showed no significant association with VTE, supported by one paper [26] for THA and three [18,26,29] for TKA.

When regarding to herbal therapy, the only one paper [26] fount it with an significantly increased risk. The paper found that the herbal therapy of traditional Thailand medicine can increase VTE risk for TKA patients, but it is uncertain whether other kinds of herbal therapy would increase the VTE risk.

### Laboratory indexes

#### Preoperative laboratory index

Two papers [35,49] examined four kinds of indexes as risk factors of VTE. One study [35] found blood glucose level ≥ 200 mg/dl with an increased VTE risk for both THA and TKA. This result is not consistent with that from papers [26,35,47,53] focusing specifically on diabetes mellitus.

Resting PaCO2 ≥ 45 mmHg has also proven to be a VTE risk factor. Resting PaO2 < 75 mmHg and RVSP ≥ 35 mmHg were reported by only one paper [49] focusing on TKA patients and showed no significant association with VTE.

### Postoperative laboratory index

Two papers [28,53] examined four risk factors of VTE for TKA alone. Each of these factors was reported with an increased VTE risk. All factors were presented in Table 7.

Higher platelet counts, hemoglobin ≧ 10.5 g/dl and AaDO2 ≧ 34 Torr were investigated by one paper [28]. All of the three laboratory indexes were collected 1 day postoperatively and were found to be VTE risk factors for TKA patients.

One articles [53] have studied the seroconvertion of IgG-class HIT (heparin-induced thrombocytopenia) antibody, an indicator of the thromboembolic omplication that can occur with heparin using. The result of this paper shows that the seroconvertion of HIT antibody can increase VTE risk of TKA patients. To our knowledge, this factor has not been studied in THA patients.

### Health care provider-related factors

#### Surgery type

Twelve papers [11-13,18,20,21,26,27,29,30,35,53] examined surgery types as risk factors of VTE. All factors reported were presented in Table 8.

TKA is a VTE risk factor, reported by two [18,35] from four papers [18,30,35,53], compared to THA. Bilateral arthroplasty surgery were reported with a significant increases VTE risk by one paper [35] for THA and two [20,35] for TKA.

One paper [41] compared the VTE rates between THA and THRA (total hip resurfacing arthroplasty) and found the former with significant increased VTE risk.

Another paper [35] for THA and two [26,35] for TKA took the revision surgery as a potential VTE risk factor, compared to primary surgery. Except for the result from one paper [26] in which the revision surgery turned out to a risk factor, other results show no significant association between the revision surgery and VTE rate.

The surgery sides of the knee, e.g. right side and left side, were also investigated by one article [26]. The result shows no significant difference between the two sides, as expected.

### Surgical technique

Five papers [12,13,18,21,29] examined surgical technics, e.g.fixation and ROBODOC milling system, as risk factors of VTE. One [18] from two papers [13,18] and two [18,21] from three papers [18,21,29] reported an increased VTE risk associated with cement fixation, for THA and TKA patients respectively, while another article [12] found ROBODOC system a protective factor compared to traditional technic.

### Operating time

Four papers [19,22,35,49] examined surgery time as a risk factor of VTE for THA or TKA. Only one paer [19] found longer surgery time (surgery lasting more than 2 hours) with increased VTE risk. Other papers which used different cut-off value, e.g. 3 hours, found no significant association between surgery time and VTE risk.

### Anesthesia

Five papers [18,21,29,30,52] examined anesthesia types as risk factors of VTE. Three papers [18,30,52] for THA and three for TKA [18,21,30] found general anesthesia with no significant association for VTE. Spinal anesthesia was reported with no significant association of TKA by one paper [29] either.

### Bleeding

One paper [28] examined bleeding volume as a risk factor of VTE for TKA and found it with an increased risk when more than 1280 ml. The bleeding volume was defined as the cumulative bleeding volume measured on the day after the surgery.

### Hospital volume

Two papers [23,55] examined lower hospital volume as a risk factor of VTE for THA and TKA.

The former article [23] which is focusing on TKA, took the hospitals with the lowest 40% surgical volume as the “low volume” ones, and those with the highest 20% surgical volume as the “high volume” hospitals, respectively. In this article, higher hospital volume was found to be VTE risk factor.

The latter article [55] applied a variety of specific cut-off value of the hospital volume, e.g. 25, 100 and 200. Significant association between higher volume and increased VTE risk was verified for THA patients, but not for TKA patients.

### Insurance type

One paper [23] examined insurance types, e.g. private insurance and Medicare/Medicaid, as risk factors of VTE for THA patients. No significant association was found in this study. Because this study was conducted in the United States, the insurance types to be investigated were based on domestic condition of US, which need to be mentioned and noticed by the readers.

### Thromboprophylaxes

#### Chemoprophylaxis

28 papers (see Table 4) examined several chemoprophylaxis schemes as risk factors of VTE for THA and TKA. Comparisons and results were listed in Table 9.

### Initiating and lasting of the prophylaxis

Two papers [21,30] examined preoperative low molecular weight heparin (LMWH) compared to postoperatively LMWH using. One paper [21] for THA and two [21,30] for TKA found the initiating time(preoperative vs. postoperative) with no significant association to VTE. Extended prophylaxis, which is defined in different way by two studies [43,57], was compared with short-duration and reported with a decreased VTE risk.

### Mechanical and physical prophylaxis

Five papers [19,24,29,30,52] examined a variety of mechanical and physical prophylaxis, e.g. stockings and early mobilization, as risk factors of VTE for THA and TKA. Earlier mobilization/ambulation was found to significantly decrease VTE risk, if achieved at either of the three time points: 24 hours after surgery, 72 hours after surgery and before discharge [19,29,30]. No significant association with VTE was found when regarding to the following comparisons: mechanical prophylaxis (vs. chemoprophylaxis) [52], below-knee stockings (vs. up-knee) [24] and weight bearing within 48 h [30].

### Confirmed factors

Risk directions of factors examined in at least three articles were included in further analysis to be confirmed, see Table 10. We found six risk VTE factors of THA and seven risk factors of TKA. Protective factors (four of THA and four of TKA) and controversial factors (four of THA and seven of TKA) were also presented in Table 10. The definition of “*confirmed*” was described in the “Analysis” section in this article.

## Discussion

We conducted a systematic review on risk factors for VTE of total joint arthroplasty including THA and TKA relating to the demographic characteristics of patients, clinical conditions and so forth. Articles published in recent ten years (2003–2013) were included with data of more than 1,150,000 patients. We presented all potential factors studied in at least one paper, and confirmed the risk directions of all factors examined in at least three papers by using “risk factor” “protective factor” and “controversial factor” as conclusions. In this systematic review of 54 high evidence level papers, six *confirmed* VTE risk factors for THA and seven for TKA were found (see Table 10).

TKA surgery were associated with higher risk of VTE than that of THA [35,65]. The better postoperative exercising of THA patients could be a reason, but the inner mechanism has not been studied and is still unclear. We suggested surgeons and physicians to give closer attention to TKA patients in monitoring of VTE.

Older age, female gender, higher BMI and bilateral surgery were found to be VTE risk factors for both THA and TKA. However, the conclusions about age and BMI apply only to particular groups of patients. Age > 75 (vs. age < 75) as well as age > 70 (vs. age < 50) proved to be a risk factor supported by some papers [30,35,53]. As for BMI, only those with BMI >30 can be taken as high VTE risk patients [19,35]. Patients with higher BMI are always associated bad hemodynamics condition which may induce the thrombogenesis. Female gender and bilateral surgery were found to be a risk factor respectively. The procoagulant function of female hormone and coagulant-response following the bilateral surgery are possible explanations [26,29].

VTE history seems to be a potential VTE risk factor with high probability. Surprisingly we found the risk to be significant only in TKA patients but not in THA patients. The medical record bias of VTE history may account for this result. The relationship between VTE history and VTE after THA/TKA deserves further and refined research.

Cemented fixation of TKA compared to cementless was found to be a risk factor for VTE in our study. Considering the inconsistent results of other studies [66,67] additional research is necessary before more definite conclusions can be drawn.

Longer surgery time was found to be a VTE risk factor for both two kinds of arthroplasty surgeries of low limb. An operation lasting more than 2 hours may increase the VTE risk, probably because of multiple surgical effects on the blood vascular system such as the endothelial injuries and hypercoagulable state [68]. However, more studies focusing on the relation between operating time and VTE rate are still needed.

Chemoprophylaxis and mechanical/physical thromboprophylaxis which have been widely used already are widely known “VTE protective factors”. A variety of comparisons between different thromboprophylaxes have been included into our systematic review (see Table 9). Enoxaparin and newly-developed direct Factor Xa inhibitors have shown great superiority to LMWH (not containing and containing enoxaparin respectively). In addition, earlier mobilization achieved at either of the three time point (see “Result” section of this article) was reported with a significant decreased risk for VTE of TKA, confirmed by several articles [19,29,30]. These conclusions undoubtedly convince us of the reasonability of thromboprophylaxis using.

Several limitations of this systematic review bear further comments as follows:

1. Definition inconsistency of “VTE” across the included papers, e.g. symptomatic or venography-proven (estimated magnitude of bias: low; effects on study results: unknown);
2. Limitation on number of available papers for each potential factor (estimated magnitude of bias: moderate; effects on study results: difficulty in assessing particular factors);
3. Selection bias of our review (only level-Iand level-II evidence were included, therefore some risk factors examined in lower level studies like case–control studies were excluded inherently) and publishing bias favoring statistically significant results (estimated magnitude of bias: moderate; effects on study results: neglect of some risk factors);
4. Confounders that have not been adjusted in studies included, e.g. mutual effects between BMI and metabolic diseases, older age and postoperative immobility (estimated magnitude of bias: moderate; effects on study results: confounding of risk factors).

In this way, the listed risk factors and protective factors of this study can only be seen as a lookup table rather than a final conclusion. Each particular potential factor need to be examined in further researches.

Doctors are nowadays facing a great challenge in preventing of VTE for total joint arthroplasty. A stratification system of VTE risk and appropriate thromboprophylaxis schemes based on risk classification which suit the circumstance of each patient are in urgent need.

Common situation is that the risk of VTE decreases accompanied with the increasing risk of bleeding when drugs were used, as a result of the dose-effect relationship of most drugs including those newly developed ones, e.g. direct factor-Xa inhibitor like TAK-442 and partial factor-VII inhibitor like TB-402 [50,56] Weitz et al. have conducted chemoprophylaxis based on their own risk classification systems, and found VTE as well as bleeding in the “high risk” group treated with high dose drugs [50,69]. It implicates that the particular dose of a drug is not enough for some patients in VTE preventing but too strong for others.

Optimal VTE prevention has not been achieved, partly because of the roughness of the existing risk stratification system. Therefore, risk stratification systems need improvement. However, there is even not any VTE risk stratification system for total joint arthroplasty, despite of the “Caprini score” [70] which is not especially for THA and TKA, to our knowledge. Further research may clarify the real VTE risk factors and develop a risk stratification system. In this way, stronger thromboprophylaxes can be given to patients of confirmed VTE risk, rather than misused to become risk factors for bleeding.

## Conclusions

This systematic review, factors which was found to be associated with VTE risk of both THA and TKA included older age, female sex, higher BMI, bilateral surgery, VTE history and surgery time > 2 hours. Cemented fixation was found to be a VTE risk factor only for TKA patients, and “TKA” itself was found to be associated with higher VTE risk, compared with THA.

Chemoprophylaxis for VTE(vs.no-prophylaxis), enoxaparin (vs.other LMWH) and direct F-Xa inhibitor (vs.LMWH) were found to be VTE protective factors for both THA and TKA. Earlier mobilization was also a protective factor for TKA. However, we can not take earlier mobilization as a VTE protective factor for THA until sufficient number of papers of high evidence level are available.

By identifying these factors, patients with relatively higher risk of VTE could be distinguished and therefore treated more intensively. Further studies are warranted to brought into more potential VTE factors to provide robust evidence for this prognostic topic.

## Abbreviations

VTE: Venous thromboenbolism
BMI: Body mass index
RA: Rheumatoid arthritis
OA: Osteoarthritis
CHF: Congesive heart failure
MI: Myocardial infarction
CKD: Chronic kidney disease
RVSP: Right ventricular systolic pressure
AaDO2: Alveolar-arterial oxygen gradient
HIT: Heparin-induced thrombocytopenia
